# Ventilator-associated respiratory infection in a resource-restricted setting: impact and etiology

**DOI:** 10.1186/s40560-017-0266-4

**Published:** 2017-12-19

**Authors:** Vu Dinh Phu, Behzad Nadjm, Nguyen Hoang Anh Duy, Dao Xuan Co, Nguyen Thi Hoang Mai, Dao Tuyet Trinh, James Campbell, Dong Phu Khiem, Tran Ngoc Quang, Huynh Thi Loan, Ha Son Binh, Quynh-Dao Dinh, Duong Bich Thuy, Huong Nguyen Phu Lan, Nguyen Hong Ha, Ana Bonell, Mattias Larsson, Hoang Minh Hoan, Đang Quoc Tuan, Hakan Hanberger, Hoang Nguyen Van Minh, Lam Minh Yen, Nguyen Van Hao, Nguyen Gia Binh, Nguyen Van Vinh Chau, Nguyen Van Kinh, Guy E. Thwaites, Heiman F. Wertheim, H. Rogier van Doorn, C. Louise Thwaites

**Affiliations:** 1 National Hospital for Tropical Diseases, Hanoi, Vietnam; 2 Oxford University Clinical Research Unit, Hanoi, Vietnam; 3 Centre for Tropical Medicine and Global Health, University of Oxford, Oxford, UK; 4 Hospital for Tropical Diseases, Ho Chi Minh City, Vietnam; 5 Bach Mai Hospital, Hanoi, Vietnam; 6 Karolinska Institutet, Stockholm, Sweden; 7 Linköping University, Linköping, Sweden; 8 University of Medicine and Pharmacy, Ho Chi Minh City, Vietnam; 9 Department of Medical Microbiology and Radboud Center for Infectious Diseases, Radboudumc, Nijmegen, Netherlands; 10 Oxford University Clinical Research Unit, Ho Chi Minh City, Vietnam

**Keywords:** Ventilator-associated respiratory infection, VARI, Ventilator-associated pneumonia, VAP, Ventilator-associated tracheobronchitis, Vat, Hospital-acquired infection, Resource-restricted, Vietnam, Carbapenem resistance, Antimicrobial resistance

## Abstract

**Background:**

Ventilator-associated respiratory infection (VARI) is a significant problem in resource-restricted intensive care units (ICUs), but differences in casemix and etiology means VARI in resource-restricted ICUs may be different from that found in resource-rich units. Data from these settings are vital to plan preventative interventions and assess their cost-effectiveness, but few are available.

**Methods:**

We conducted a prospective observational study in four Vietnamese ICUs to assess the incidence and impact of VARI. Patients ≥ 16 years old and expected to be mechanically ventilated > 48 h were enrolled in the study and followed daily for 28 days following ICU admission.

**Results:**

Four hundred fifty eligible patients were enrolled over 24 months, and after exclusions, 374 patients’ data were analyzed. A total of 92/374 cases of VARI (21.7/1000 ventilator days) were diagnosed; 37 (9.9%) of these met ventilator-associated pneumonia (VAP) criteria (8.7/1000 ventilator days). Patients with any VARI, VAP, or VARI without VAP experienced increased hospital and ICU stay, ICU cost, and antibiotic use (*p* < 0.01 for all). This was also true for all VARI (*p* < 0.01 for all) with/without tetanus. There was no increased risk of in-hospital death in patients with VARI compared to those without (VAP HR 1.58, 95% CI 0.75–3.33, *p* = 0.23; VARI without VAP HR 0.40, 95% CI 0.14–1.17, *p* = 0.09). In patients with positive endotracheal aspirate cultures, most VARI was caused by Gram-negative organisms; the most frequent were *Acinetobacter baumannii* (32/73, 43.8%) *Klebsiella pneumoniae* (26/73, 35.6%), and *Pseudomonas aeruginosa* (24/73, 32.9%). 40/68 (58.8%) patients with positive cultures for these had carbapenem-resistant isolates. Patients with carbapenem-resistant VARI had significantly greater ICU costs than patients with carbapenem-susceptible isolates (6053 USD (IQR 3806–7824) vs 3131 USD (IQR 2108–7551), *p* = 0.04) and after correction for adequacy of initial antibiotics and APACHE II score, showed a trend towards increased risk of in-hospital death (HR 2.82, 95% CI 0.75–6.75, *p* = 0.15).

**Conclusions:**

VARI in a resource-restricted setting has limited impact on mortality, but shows significant association with increased patient costs, length of stay, and antibiotic use, particularly when caused by carbapenem-resistant bacteria. Evidence-based interventions to reduce VARI in these settings are urgently needed.

**Electronic supplementary material:**

The online version of this article (10.1186/s40560-017-0266-4) contains supplementary material, which is available to authorized users.

## Background

Ventilator-associated respiratory infection (VARI) is the commonest hospital-acquired infection in intensive care units (ICUs) [1, 2]. The condition includes both ventilator-associated tracheobronchitis (VAT) and ventilator-associated pneumonia (VAP) and has an incidence in resource-rich countries of 1–22 per 1000 ventilator days [3–6]. One reason for the variable incidence is the use of differing definitions, and there is increasing evidence to suggest that current definitions do not correlate well with real-world clinical practice [7–10].

In high-income settings, VARI has consistently been shown to be associated with increased hospital costs and length of ICU stay [11, 12], but estimating mortality attributable to VARI is difficult as patients requiring longer periods of ventilatory support are often the most severely ill patients and at increased risk of death. In an attempt to overcome this, a recent individual patient meta-analysis showed that the attributable mortality due to VAP was mainly associated with intermediate-severity underlying disease [13]. The relationship of VAT and mortality is even less clear, with some studies showing no deleterious effect on mortality [5, 14]. Nevertheless, a survey of physicians from 288 ICUs showed that 50% of physicians considered VAT to be associated with increased mortality [4].

There are fewer data concerning the occurrence of VARI in resource-restricted settings but it appears to be at least as common, if not more so, than in resource-rich settings [15–20]. However, estimating its true incidence is difficult and current surveillance definitions have been criticized as particularly inapplicable in resource-restricted settings [21]. VARI occurring in these locations is generally assumed to have similar impact to that occurring in high-income countries, but there are limited data to confirm this, and some studies have indicated significantly worse outcomes [17, 22]. Differences in underlying diagnoses, age, comorbidity, and nutritional status may all influence outcome in addition to variations in treatment facilities and staffing levels [23]. Studies suggest that in low- and middle-income countries (LMICs), infections are usually caused by multidrug-resistant Gram-negative organisms [24, 25]. Some studies, although not all, suggest poorer outcome in these patients [17, 26, 27]. This combination of high rates of incidence and antibiotic resistance means VARI may also have an important influence on antibiotic use. A recent study from Vietnam reported that VARI is the most common reason for antibiotic use in ICU [16].

Understanding the nature and impact of VARI in low-resource settings is especially important as these are places where implementation of relatively simple preventative interventions could have a significant impact [17, 23]. In many ICUs, high rates of carbapenem-resistant pathogens necessitate increasing use of empiric regimes including colistin [16]. Identifying factors associated with antimicrobial resistance would therefore enable more effective prevention and treatment measures [17].

In this study, we performed a multicenter prospective study following patients receiving mechanical ventilation in four intensive care units in Vietnam with the aim of providing a comprehensive description of VARI in a resource-restricted setting. We examine the incidence, etiology, and outcome in addition to antibiotic usage and antimicrobial resistance to provide a complete clinical picture of VARI and its impact.

## Methods

This was a prospective observational study performed in four intensive care units of three tertiary referral hospitals in Vietnam between November 2013 and November 2015 (total 79 ICU beds). Three sites were ICUs in two specialist infectious disease hospitals: one serving northern Vietnam and the other southern Vietnam (combined capacity 800 beds). The fourth site was a mixed medical and surgical ICU of a 1900-bed teaching hospital. Patients were eligible for study entry if ≥ 16 years old and expected to be mechanically ventilated for at least 48 h. Patients intubated for more than 48 h prior to ICU admission were excluded. An initial sample size of 600 was calculated based on previous data to estimate VAP incidence of 30% with 95% confidence interval of 3.7% [16]. However, due to slow recruitment rate, recruitment was stopped after 2 years and enrollment of 450.

Baseline demographic and clinical data were recorded on admission to the study, and an endotracheal aspirate was taken as previously described [28]. Patients were followed for 28 days for development of VARI in two sites and until extubation in the remaining sites. Hospital outcome was recorded (death or discharge/transfer). Clinically suspected VAP was investigated with chest X-ray and endotracheal aspirate. VAP was diagnosed according to modified US Centers for Disease Control and Prevention (US CDC) criteria [29–31] as follows. Firstly, a deterioration in ventilation following a period of stability defined according to positive end expiratory pressure (PEEP): ≥ 2 days of stable or decreasing daily minimum PEEP followed by a rise in daily minimum PEEP of ≥ 3 cm H_2_O, sustained ≥ 2 calendar days; or F_i_O_2_: ≥2  days of stable or decreasing daily minimum F_i_O_2_ followed by a rise in daily minimum F_i_O_2_ ≥ 0.15 points, sustained ≥ 2 calendar days. Secondly, systemic signs of fever > 38 °C or < 36 °C or white blood cell count > 12 × 10^9/^L or < 4 × 10^9^/L were required. Final criteria were increased/new purulent tracheobronchial secretions or ≥ 25 neutrophils per low power field (10 objective) on Gram stain of endotracheal aspirate and either new and persistent infiltrates, consolidation, or cavitation as read by two study physicians on chest X-ray, or the decision to commence new antibiotic therapy. VARI was defined as including those patients with VAP (defined as above) and patients meeting the following criteria (modified from Craven et al. [8]): clinically increased sputum volume or purulent sputum by microscopic examination (≥ 25 neutrophils per low power filed on Gram stain of endotracheal aspirate) and either systemic signs of infection (fever or white blood count as above) in addition to the clinician starting antibiotics within 2 days of these features developing. Thus, for the purposes of this paper, we use the term “VAP” to refer to patients fitting the above definition of VAP, “VARI” to describe all patients with either VAP or VARI as defined above, and “VARI without VAP” to refer to patients with VARI as above but not meeting full criteria for VAP. It can be considered that VARI would therefore encompass all patients with VAP and ventilator-associated tracheobronchitis (VAT).

The microbiological cause of VARI was determined by isolating at least one pathogenic organism from blood culture or from endotracheal aspirate ≥ 10^5^ colony forming units/ml or equivalent semi-quantitative culture. Microbiological methods varied by site in line with routine clinical microbiological work. Briefly, in all sites, the BACTEC (Becton Dickinson, Sparks, MD, USA) automated system for blood cultures was used with a single aerobic culture bottle inoculated in all suspected cases. Endotracheal aspirate samples were subjected to Ziehl-Neelsen and Gram staining prior to incubation on sheep blood in blood agar base (BioMérieux, Marcy l’Étoile, France), MacConkey, and chocolate blood agar. Colonies were identified using routine biochemical testing (involving VITEK2 and/or API test (BioMérieux) or MALDI-TOF MS (Bruker Daltonics, Bremen, Germany)). Antimicrobial susceptibility testing was carried out by the Kirby/Bauer disc diffusion methods, including double disk diffusion for detection of extended spectrum beta-lactamases (ESBL), using cutoffs as per CLSI 2012 guidelines or using VITEK2 depending on site. In addition, CHROMagar (CHROMagar, Paris, France) for detection of methicillin-resistant *Staphylococcus aureus* (MRSA), ESBLs, *Amp*C beta-lactamase, and KPC carbapenemase were used in some sites.

Data were recorded onto a case record form and entered into a secure database. Data analysis used Stata Statistical Software, release 8 (Stata Corp LP, College Station, TX, USA). Data are given as median (interquartile range (IQR)). Comparison of medians was performed using Mann–Whitney *U* test. Tests of proportion of categorical variables were carried out using chi-squared test. Days at risk of VARI were calculated as days of ventilation and limited to 28 days after enrollment for those not followed for VARI after 28 days. Risk of in-hospital death following VARI was assessed using Cox’s proportional hazards model; those followed for VARI only until 28 days after enrollment were censored at 28 days. Patients with missing hospital discharge data were excluded from this analysis; those with missing APACHE_II data were allocated mean scores depending on admission diagnosis. Logistic regression models were used to evaluate the relationship between admission diagnosis and risk of VARI and relationship with antibiotic treatment. Relationship of ventilation length and admission diagnosis was assessed using Mann–Whitney *U* test.

Antibiotic use was assessed using days of therapy (DOT) defined as the sum of all days on each antibiotic per 100 patient days. For the purpose of this analysis, only antibiotics for systemic use were included (i.e., topical preparations were not) and both metronidazole, which is used for treatment of tetanus, and anti-tuberculosis medications were excluded. Antibiotic therapy was defined as “adequate” if the pathogen showed in vitro susceptibility and “inadequate” if it showed intermediate or resistant results. Samples with no significant growth were excluded from these analyses. Cox models assessing effect of antibiotic resistance or adequacy of empiric therapy were constructed using time from VARI as time-to-event variable.

Economic analysis was limited to direct medical costs, derived from hospital bills and based on fees charged to the National Health Insurance and to the patients themselves combined.

## Results

Four hundred fifty patients were enrolled between November 2013 and November 2015. One patient withdrew from the study and 75 patients were excluded from analyses: 69 were ventilated for less than 48 h and 6 patients were transferred for extra-corporeal membrane oxygenation (ECMO). Thus, data from 374 patients were available for analysis.

A total of 37/374 (9.9%) patients were diagnosed with VAP with a total incidence density of 8.7/1000 ventilator days. A further 55/374 cases (14.7%), not fulfilling VAP criteria, were diagnosed with VARI, giving a total of 92/374 (24.6%) cases of VARI and an incidence density of 21.7/1000 ventilator days for all VARI. Median time from start of ventilation to developing VAP was 10 days (IQR 5.5–12) and 9 days (IQR 6–15) for cases of VARI without VAP.

### Risk factors for VARI

Baseline data for patients are given in Table 1. Baseline data by site are provided in Additional file 1: Table S1. Patients with both VAP and VARI without VAP experienced longer durations of mechanical ventilation than patients with no VARI. Median duration of ventilation for those with VAP was 22 days (IQR 17–31), 21 days with VARI without VAP (IQR 14–28 days), compared to 10 days (IQR 6–16) with no VARI (*p* < 0.01 for both) (Table 2 and Additional file 1: Table S2).

**Table 1:** Baseline characteristics of patients with and without VARI

	All VARI (*n* = 92)	VAP (*n* = 37)	VARI without VAP (*n* = 55)	No VARI (*n* = 286)
Age	48 (36–61.5)	46 (37–62)	50 (35–62)	53 (40–65)
M:F	70:22	25:12	45:10	197:85
SOFA	3 (2–6)	5 (2–8)	3 (1–3)	4 (3–7)
APACHE	8.5 (5–14)	11 (5–15)	7 (5–14)	11 (7–15)
BMI	21.97 (19.72–24.56)	22.12 (21.12–24.61)	21.97 (19.03–23.53)	20.56 (18.69–23.48)
Comorbidity	26 (28.3%)	14 (37.9%)	12 (21.8%)	118 (42.1%)
Hospital previous 90 days†	7 (7.6%)	3 (8.1%)	4 (7.3%)	34 (12.1%)
Transferred for this illness	69 (75%)	27 (73.0%)	42 (76.4%)	212 (75.18)
Antibiotics in last 90 days	4 (4.4%)	3 (8.1%)	1 (1.8%)	21 (7.5%)
CNS infection	11 (12.0%)	1 (2.7%)	10 (18.2%)	49 (17.4%)
Dengue	3 (3.3%)	2 (5.4%)	1 (1.8%)	6 (2.1%)
Sepsis/septic shock	11 (12.0%)	6 (16.2%)	5 (9.1%)	44 (15.6%)
Tetanus	48 (52.2%)	14 (37.8%)	34 (61.8%)	84 (29.8%)
Pneumonia	7 (7.6%)	7 (18.9%)	0 (0%)	66 (23.4%)

Values given are median (IQR) or count (percent)

†*n* = 249

**Table 2:** Outcome of patients with VARI, VAP, other VARI, and no VARI

	All VARI (*n* = 92)	VAP (*n* = 37)	VARI without VAP (*n* = 55)	No VARI (*n* = 286)
ICU length of stay (days)	27 (21–37)	25 (19–37)	27 (25–38)	16 (10–28)
Days ventilated (days)	22 (15.5–29.5)	21 (14–28)	22 (17–31)	10 (6–16)
ICU cost (USD) *	4723 (2778–7783)	7213 (3011–8329)	4196 (2726–7193)	2534 (1493–4407)
Hospital length of stay (days)	38 (26–49)	31 (22–46)	39.5 (29.5–49)	25 (14–39)
Hospital cost (USD)**	4434 (2656–7822)	4522 (2716–7990)	4167 (2658–6985)	2639 (1555–4576)
Antibiotics use (DOT)	31 (18–42.5)	35 (21–45)	30 (17–38)	18 (10–28)
Antibiotics in hospital before VARI	73/92 (79.4%)	28/38 (75.6%)	45/55 (87.7%)	255/282 (90.43%)
In-hospital mortality	17/91 (18.7%)	13/37 (35.1%)	4/54 (7.4%)	54/277 (19.5%)

Values given are median (IQR) or count (percent)

* *n* = 372; ** *n* = 313

To identify baseline predictors of all VARI, admission diagnosis was examined. As this was significantly associated with risk of VARI (OR 1.23 95% CI 1.05–1.45 *p* = 0.01), different admission diagnoses were investigated. Patients with community-acquired pneumonia had the lowest risk of VARI with a risk of 9.6% (95% CI 3–17%). Compared to these, there was a significantly increased risk of VARI in patients with an admission diagnosis of tetanus (36.4%, 95% CI 28.2–45.2%, *p* < 0.01) or “other diagnosis” category (26.7%, 95% CI 14.6–41.9%, *p* = 0.02) (Additional file 1: Table S3). Those with tetanus also had significantly longer duration of mechanical ventilation at a median 18 (IQR 13–24) days and lowest antibiotic use on admission compared to those with community-acquired pneumonia (*p* < .01 both) (Additional file 1: Table S4).

### Impact of VARI on outcomes

Patients with VARI experienced longer hospital and ICU stay, increased hospital, and ICU cost and increased antibiotic use in the first 28 days of study. This was true for both VAP and VARI without VAP (Table 2) (*p* < 0.01 all).

Compared to patients with no VARI, there were no significant differences in risk of in-hospital death with VARI (HR 0.88, 95% CI 0.44–1.73, *p* = 0.70). When VAP and VARI without VAP subgroups were analyzed, there was a trend towards higher risk of in-hospital death in patients with VAP compared to those with no VARI (HR 1.58, 95% CI 0.75–3.33, *p* = 0.23). However, there was a lower risk of in-hospital death in patients with VARI without VAP compared to those with no VARI (HR 0.40, 95% CI 0.14–1.17, *p* = 0.09) which was non-significant. After correction for APACHE II scores, these trends remained non-significant: VAP HR 1.67, 95% CI 0.78–3.55, *p* = 0.18 and VARI without VAP HR 0.46, 95% CI 0.15–1.39, (*p* = 0.17).

Due to the high proportion of patients with tetanus and its possible confounding, the effect of tetanus on the impact of VARI was specifically examined. Similar to the whole-sample analysis, in both patients with and without tetanus, patients with VARI experienced increased hospital and ICU length of stay, increased duration of ventilation, increased hospital and ICU costs, and increased antibiotic use within the 28-day study period (Additional file 1: Tables S5 and S6.). When tetanus status, Apache II, and interaction between tetanus and VARI were included in the cox model, there were no differences in the risk of in-hospital death following either VAP or other VARI (VAP HR 1.79, 95% CI 0.84–3.82, *p* = 0.13; VARI without VAP HR 0.43, 95% CI 0.13–1.38, *p* = 0.15).

### Microbiology of VARI

Of the 92 patients treated for VARI, 77 had endotracheal aspirates taken within 48 h of VARI diagnosis, 73 of which had positive cultures (Table 3). The predominant bacterium was *Acinetobacter baumannii* (32 patients) followed by *Klebsiella pneumoniae* (26 patients) and *Pseudomonas aeruginosa* (24 patients). 68/73 (92%) patients had specimens containing one or more of these three organisms.

**Table 3:** Organisms isolated from initial endotracheal aspirates within 48 h of VARI diagnosis

	*N* (% isolates)	Carbapenem resistance *N* (%)
*Acinetobacter baumannii*	32 (29.7%)	27/32 (84.4%)
*Klebsiella pneumoniae*	26 (24.1%)	6/26 (23.1%)
*Pseudomonas aeruginosa*	24 (22.2%)	11/24 (45.8%)
*Staphylococcus aureus*	10 (9.3%)	5* (50%)
*Haemophilus influenza*	5 (4.6%)	0 (0%)
*Elizabethkingia meningoseptica*	3 (2.8%)	1 (33.3%)
*Stenotrophomonas maltophilia*	3 (2.8%)	3 (100%)
*Streptococcus pneumoniae*	3 (2.8%)	0* (0%)
*Escherichia coli*	1 (0.9%)	1 (100%)
*Proteus mirabilis*	1 (0.9%)	0 (0%)

*Carbapenem susceptibility not formally assessed but carbapenem susceptibility assumed from penicillin or methicillin sensitivity

### Antibiotic resistance and impact on outcomes

New antimicrobial therapy given within 48 h of VARI diagnosis is given in Additional file 1: Table S7. The majority of cases (49/92, 54.3%) were treated with a carbapenem; colistin was used in 25/92 (27.2%) cases and combination therapy was used in 36/92 (39.1%) cases. Adequacy of empiric initial antimicrobial therapy of VARI could be assessed in 71 cases and was inadequate in 20/71 (28%) cases. However, inadequate therapy was not associated with increased mortality (HR 0.1.11, 95% CI 0.34–3.66, *p* = 0.86), nor were there differences in length of ICU stay or ICU costs (Table 4). Of note, all of the specimens from patients treated with inadequate antibiotic contained one or more carbapenem-resistant bacteria.

**Table 4:** Adequacy of initial empiric antibiotic therapy for VARI

	Adequate	Inadequate	*p*
In-hospital mortality	9/51	4/20	1.00
ICU length of stay (days)	27 (22–37)	27.5 (25–37)	0.74
Hospital length of stay (days)	38 (26–49)	39 (28.5–44)	0.89
ICU cost (USD)	4797 (2726–7806)	5485 (3541–7259)	0.88
Hospital cost (USD)	4461 (2666–7883)	5549 (3843–7543)	0.54

Values given are median (IQR) or count (percent)

5/10 (50%) *Staphylococcus aureus* isolates were methicillin-resistant. 11/24 (46%) of *P. aeruginosa* isolates and 27/32 (84%) *A. baumannii* isolates were resistant to carbapenems, compared to only 6/26 (23%) of *K. pneumoniae*. In total, 40/68 (59%) patients with positive culture for *P. aeruginosa*, *A. baumannii*, or *K. pneumoniae* had carbapenem-resistant isolates. Patients with VARI attributed to these carbapenem-resistant bacteria had significantly greater ICU costs than patients with non-resistant isolates (6053 USD (IQR 3807–7824) vs 3131 USD (IQR 2109–7552), *p* = 0.04). There were no differences between length of hospital or ICU stay but patients with carbapenem-resistant isolates had increased antibiotic use during the study period (37.5 (IQR 29.0–45.5) days of therapy (DOT) compared to 21 (IQR 15.5–33.0) DOT, *p* < 0.01) (Table 5). Additionally, patients with carbapenem-resistant isolates showed a trend towards increased in-hospital death (HR 2.82, 95% CI 0.75–6.75, *p* = 0.15) which remained when adequacy of initial empiric antibiotic therapy and APACHE II scores were taken into account (HR 2.82, 95% CI 0.87–9.19, *p* = 0.09).

**Table 5:** Impact of carbapenem-resistant bacteria causing VARI

	Carbapenem-resistant Enterobacteriaceae/*A. baumannii* or *P. aeruginosa*	Carbapenem-sensitive Enterobacteriaceae/*A. baumannii* or *P. aeruginosa*	*p*
ICU length of stay (days)	26 (20.5–33.5)	29.5 (23.5–39.5)	0.23
ICU cost (USD)	6053 (3806–7824)	3131 (2108–7551)	0.04
In-hospital mortality	10/40 (25%)	3/28 (10.7%)	0.14
Antibiotic use (DOT)	37.5 (29–45.5)	21 (15.5–33)	<0.01

Values given are median (IQR) or count (percent)

In order to assess potential risk factors for development of VARI with carbapenem-resistant bacteria, treatment with carbapenems in ICU before diagnosis of VARI was examined. 22/39 (56%) patients with carbapenem-resistant isolates of *P. aeruginosa*, *A. baumannii*, or *K. pneumoniae* were treated with carbapenems between ICU admission and development of VARI, compared to only 2/26 (7.7%) of those with sensitive isolates (*p* < 0.01).

## Discussion

This study has shown that VARI is a common and important problem in resource-restricted ICUs and patients with VARI have an increased length of ICU stay, ICU cost, and antibiotic use. Calculating attributable mortality due to VARI is difficult due to difficulty identifying a control group and the numerous possible confounders. By using survival analysis, we aimed to account for differences in time at risk. In addition, by including a large subgroup of patients with tetanus, we have gained a unique insight into the impact of VARI on patient outcome. Patients with tetanus have little comorbidity or organ dysfunction in addition, and they lack features such as increased pulmonary vascular permeability that may confuse the diagnosis of VARI. In these patients, we observed increased risk of VARI but very low mortality, and there was no evidence that risk of in-hospital death following VARI differed between patients with or without tetanus.

Although we did not observe any increased risk of in-hospital death in patients with either VAP or VARI without VAP, there was a trend towards improved outcome in patients with VARI without VAP and worse outcome in VAP. Similar findings have previously been reported in different populations. A large study of 2960 patients in Europe and South America reported mortality rates of 40% with VAP, 29% with VAT, and 30% with no VARI [5]. Similarly, a smaller observational study of patients in a single centre in France reported a mortality rate of 29% in VAT patients compared to 36% of controls without VARI [14]. VARI may be viewed as a continuum between VAT and VAP both in terms of severity and chronology: i.e., there is a progression from asymptomatic bacterial colonization of the respiratory tract through tracheobronchitis and eventually to pneumonia when chest X-ray changes become apparent [8, 32]. It is possible that patients meeting the full criteria for VAP have more severe disease, are diagnosed later, and therefore have worse outcome. This is supported by recent evidence that patients with VAP treated early with antibiotics showed higher response rates [33].

In 2013, as this study was being conceived, the CDC introduced a system of ventilator-associated event definitions, which encompassed criteria for ventilator-associated pneumonia. Our choice of definitions for use in this study was influenced by a desire to balance relevant, objective definitions with these new definitions. These definitions have now been widely criticized, and it has been recognized that they may need to be adjusted for specific patient groups [34]. The CDC explicitly states that some elements of the new framework (probable VAP) are not suitable for public reporting or bench-marking [35]. For this reason, we chose to use the definitions we felt best reflected clinical practice and were relevant to the main issues of this study, namely the high use of broad-spectrum antibiotics in resource-restricted ICUs and outcome of patients treated for VARI. By using clear and consistent definitions throughout, we aimed to minimize bias when comparing these outcomes. As our main aim was to describe actual practice in resource-limited ICUs, and three out of four participating ICUs in our study were specialist infectious disease units, we deliberately chose to include patients with infections, including an admission diagnosis of pneumonia. An earlier pilot study had shown that not including patients with a possible pneumonia on admission led to patients with other primary diagnoses such as sepsis or meningitis being excluded from the study due to abnormal baseline chest X-rays. In view of this, we felt excluding these patients would introduce significant bias and reduce the relevance of this study for future practice.

Overall, compared to other studies in resource-restricted settings, we recorded a low incidence of VAP, but comparable levels of total VARI [16, 26, 36]. The low rate of VAP may be due to the insensitivity of VAP criteria used in our setting resulting in possible misclassification of cases. In our ICUs, low staffing levels, lack of ICU-specific training, and less frequent monitoring mean that higher ventilator settings are often used and ventilator settings are infrequently changed to reduce the risk of hypoxia. Thus, the demonstrable deterioration in ventilator settings (i.e., PEEP or F_i_O_2_) to diagnose VAP may be less likely to occur resulting in possible VAP cases classified as VARI without VAP. However, a strength of our study is that we have included all patients with VARI, thus patients not meeting VAP criteria, but treated for ventilator-related respiratory infection, have still been included in the analyses. The differences in VAP rates between ICUs included in our study may reflect differences in practices between ICUs as the overall VARI rates are similar.

Unlike VARI described in high-income settings, isolates associated with VARI were dominated by Gram-negative bacteria [37]. Of note, 92% of our patients had VARI attributable to one or more of the World Health Organization’s three “critical” priority organisms: *K. pneumoniae*, *P. aeruginosa*, and *A. baumannii* [38]. We report high levels of antimicrobial resistance among these and also note that resistance to carbapenems was associated with a worse outcome and that prior use of carbapenems during hospital admission was a risk factor for isolation of carbapenem-resistant bacteria, suggesting a role for carbapenem-sparing agents in the management of severe infection. Furthermore, carbapenem resistance had a significant and substantial impact on costs and antibiotic use.

The lack of correlation between inadequate empiric therapy and mortality is in keeping with some of the literature from high-income countries, much of which comes from studies in sepsis and septic shock, yet is out of step with other studies [38–40]. In the context of this study, it most likely also relates to the behavior of clinicians faced with a single remaining option of colistin for treatment of increasingly resistant Gram-negative infections, reserving empiric treatment more likely to be adequate (i.e., colistin) for more severe cases. We have only analyzed the effects of adequate and inadequate therapy with respect to initial empiric therapy, and it is also possible that timely reporting of microbiological culture results enabled rapid rectification of inadequate regimes, hence limited impact on outcome measures.

Patient hospitalization costs were significantly and substantially higher in patients with VARI and approximately double for those with carbapenem-resistant organisms. In our setting, antibiotics such as carbapenems or colistin form a higher proportion of ICU cost than in resource-rich settings and treatment regimens for multidrug-resistant organisms include treatment with more expensive antibiotics for longer periods. A limitation of our study is that we have not been able to divide healthcare costs before and after VARI and as such may be subject to immortal time bias. Furthermore, we have only analyzed direct patient treatment costs and have not taken account of additional healthcare costs of staffing and equipment or indirect costs such as lost earnings, travel, and subsistence costs of patients and family members providing care. In a resource-restricted setting such as ours, where much of hospital care is provided by family members, these costs are likely to be substantial and an important additional burden.

Despite these limitations, we believe VARI remains an important problem in resource-restricted ICUs and clinicians working in these settings need locally relevant evidence demonstrating the efficacy and safety of interventions to reduce its incidence. Previous work aiming to prevent infection through patient positioning and hand hygiene have not shown benefit in our setting [24, 41], emphasizing the need for locally derived data. To this end, we are engaged in a randomized controlled trial of continuous cuff pressure to prevent ventilator-associated respiratory infections (ClinicalTrials.gov idn NCT02966392).

## Conclusions

VARI is a significant problem in a resource-restricted ICU setting. Despite no observed impact on mortality, there are significant and substantial cost implications related to occurrence of VARI, particularly that associated with carbapenem-resistant bacteria. Dealing with these high rates of carbapenem resistance needs to involve both antibiotic stewardship and infection prevention strategies.

## Additional file

Additional file 1: Table S1. Baseline characteristics by site. **Table S2.** VARI and outcome by site. **Table S3.** Risk of VARI according to admission diagnosis. **Table S4.** Duration of ventilation and antibiotic use in patients according to admission diagnosis. **Table S5.** Impact of VARI in patients with and without tetanus. **Table S6.** Impact of VARI and other VARI in patients with and without tetanus. **Table S7.** Empiric antibiotic therapy for VARI (DOCX 16 kb)

## Abbreviations

CI: Confidence interval
DOT: Days of therapy
ECMO: Extra-corporeal membrane oxygenation
ESBL: Extended spectrum beta lactam
FiO2: Fraction of inspired oxygen
HR: Hazard ratio
ICU: Intensive care unit
IQR: Interquartile range
LMIC: low- and middle-income country
PEEP: Positive end expiratory pressure
USD: United States dollar
VAP: Ventilator-associated pneumonia
VARI: Ventilator-associated respiratory infection
VAT: Ventilator-associated tracheobronchitis
