# Supplementary material for: Ventilator-associated respiratory infection in a resource-restricted setting: impact and etiology
Source: J Intensive Care. 2017 Dec 19;5:69. doi: 10.1186/s40560-017-0266-4 (PMC5738227; doi:10.1186/s40560-017-0266-4)
Supplement: Additional file 1: Table S1. — Baseline characteristics by site. Table S2. VARI and outcome by site. Table S3. Risk of VARI according to admission diagnosis. Table S4. Duration of ventilation and antibiotic use in patients according to admission diagnosis. Table S5. Impact of VARI in patients with and without tetanus. Table S6. Impact of VARI and other VARI in patients with and without tetanus. Table S7. Empiric antibiotic therapy for VARI (DOCX 16 kb) [file 40560_2017_266_MOESM1_ESM.docx]

Ventilator Associated Respiratory Infection in a Resource-Restricted Setting: Impact and Etiology

Vu Dinh Phu* ^1,2^, Behzad Nadjm*^2,3^, Nguyen Hoang Anh Duy^4^, Dao Xuan Co^5^, Nguyen Thi Hoang Mai^2,4^, Dao Tuyet Trinh^1^, James Campbell^2,3^, Dong Phu Khiem^1^, Tran Ngoc Quang^1^, Huynh Thi Loan^4^_,_ Ha Son Binh^5^, Quynh-Dao Dinh^2^, Duong Bich Thuy^2,4^, Huong Nguyen Phu Lan^2,4^, Nguyen Hong Ha^1^, Ana Bonell^2^, Mattias Larsson^6^, Hoang Minh Hoan^5^, Đang Quoc Tuan^5^, Hakan Hanberger^7^, Hoang Nguyen Van Minh^3^, Lam Minh Yen^2^, Nguyen Van Hao^4,8^, Nguyen Gia Binh^5^, Nguyen Van Vinh Chau^4^, Nguyen Van Kinh^1^, Guy E Thwaites^2,3^, Heiman F Wertheim^9^, H Rogier van Doorn^2,3^, C Louise Thwaites^2,3^

* Joint first authors

Affiliations

1. National Hospital for Tropical Diseases, Hanoi, Viet Nam
2. Oxford University Clinical Research Unit, Viet Nam
3. Centre for Tropical Medicine and Global Health, University of Oxford, UK
4. Hospital for Tropical Diseases, Ho Chi Minh City, Viet Nam
5. Bach Mai Hospital, Hanoi, Viet Nam
6. Karolinska Institutet, Stockholm, Sweden
7. Linköping University, Linköping, Sweden
8. University of Medicine and Pharmacy, Ho Chi Minh City, Viet Nam
9. Department of Medical Microbiology and Radboud Center for Infectious Diseases, Radboudumc, Nijmegen, Netherlands

Email Addresses:

Vu Dinh Phu: vudinhphu07@gmail.com

Behzad Nadjm: bnadjm@oucru.org

Nguyen Hoang Anh Duy: nguyenhoanganhduy2009@gmail.com

Dao Xuan Co^:^ daoxuanco@gmail.com

Nguyen Thi Hoang Mai: mainth@oucru.org

Dao Tuyet Trinh: tuyettrinhxn@gmail.com

James Campbell: jcampbell@oucru.org

Dong Phu Khiem: drkhiemnhtd@gmail.com

Tran Ngoc Quang: ngoc_quang_tht@yahoo.com

Huynh Thi Loan^:^ loansuv@gmail.com

Ha Son Binh^:^ hasonbinhbs@gmail.com

Quynh-Dao Dinh: queenie.dinh@gmail.com

Duong Bich Thuy: duongicu@gmail.com

Huong Nguyen Phu Lan: lannph@oucru.org

Nguyen Hong Ha: dr_hongha@yahoo.com

Ana Bonell: anabonell@yahoo.co.uk

Mattias Larsson: mattias.larsson@ki.se

Hoang Minh Hoan: minhhoandttc@gmail.com

Đang Quoc Tuan: dangquoctuan.hstc@gmail.com

Hakan Hanberger: hakan.hanberger@liu.se

Hoang Nguyen Van Minh: hoangnvm@oucru.org

Lam Minh Yen: yenlm@oucru.org

Nguyen Van Hao: haodiep61@gmail.com

Nguyen Gia Binh: drbinhicu@gmail.com

Nguyen Van Vinh Chau: chaunvv@oucru.org

Nguyen Van Kinh: kinhnv@nhtd.vn

Guy E Thwaites: gthwaites@oucru.org

Heiman F Wertheim: Heiman.wertheim@gmail.com

H Rogier van Doorn: rvandoorn@oucru.org

C Louise Thwaites: ltwhaites@oucru.org

Corresponding author: C Louise Thwaites: lthwaites@oucru.org

Abstract (341 words)

Background: Ventilator associated respiratory infection (VARI) is a significant problem in resource-restricted ICUs, but differences in case-mix and etiology means VARI in resource-restricted intensive care units (ICUs) may be different from that found in resource-rich units. Data from these settings are vital to plan preventative interventions and assess their cost-effectiveness, but few are available.

Methods: We conducted a prospective observational study in 4 Vietnamese ICUs to assess the incidence and impact of VARI. Patients ≥16 years old and expected to be mechanically ventilated >48 hours were enrolled in the study and followed daily for 28 days following ICU admission.

Results: 450 eligible patients were enrolled over 24 months and after exclusions, 374 patients’ data were analysed. A total of 92/374 cases of VARI (21.7/1000 ventilator-days) were diagnosed; 37 (9.9%) of these met ventilator associated pneumonia (VAP) criteria (8.7/1000 ventilator-days). Patients with any VARI, VAP or VARI without VAP experienced increased hospital and ICU stay, ICU cost and antibiotic use (p<0.01 for all). This was also true for all VARI (p<0.01 for all) with/without tetanus. There was no increased risk of in-hospital death in patients with VARI compared to those without (VAP: HR 1.58, 95% CI 0.75-3.33, p=0.23; VARI without VAP HR 0.40, 95% CI 0.14-1.17, p=0.09. In patients with positive endotracheal aspirate cultures, most VARI was caused by Gram-negative organisms; the most frequent were *Acinetobacter baumannii* (32/73, 43.8%) *Klebsiella pneumoniae* (26/73, 35.6%) and *Pseudomonas aeruginosa* (24/73, 32.9%). 40/68 (58.8%) patients with positive cultures for these had carbapenem-resistant isolates. Patients with carbapenem-resistant VARI had significantly greater ICU costs than patients with carbapenem-susceptible isolates (6053 USD (IQR 3806-7824) vs 3131 USD (IQR 2108-7551), p=0.04) and after correction for adequacy of initial antibiotics and APACHE II score, showed a trend towards increased risk of in-hospital death ((HR 2.82, 95% CI 0.75-6.75, p=0.15).

Conclusions: VARI in a resource-restricted setting has limited impact on mortality, but shows significant association with increased patient costs, length of stay and antibiotic use, particularly when caused by carbapenem-resistant bacteria. Evidence-based interventions to reduce VARI in these settings are urgently needed.

Keywords: Ventilator associated respiratory Infection, VARI, Ventilator associated pneumonia, VAP, Ventilator associated tracheobronchitis, VAT, Hospital acquired infection. Resource-restricted, Viet Nam, Carbapenem-resistance, antimicrobial-resistance

Background

Ventilator associated respiratory infection (VARI) is the commonest hospital acquired infection in intensive care units (ICUs) [1, 2]. The condition includes both ventilator associated tracheobronchitis (VAT) and ventilator associated pneumonia (VAP) and has an incidence in resource-rich countries of 1-22 per 1000 ventilator days [3–6]. One reason for the variable incidence is the use of differing definitions and there is increasing evidence to suggest that current definitions do not correlate well with real-world clinical practice [7–10].

In high-income settings, ventilator associated respiratory infection (VARI) has consistently been shown to be associated with increased hospital costs and length of ICU stay [11, 12], but estimating mortality attributable to VARI is difficult as patients requiring longer periods of ventilatory support are often the most severely ill patients and at increased risk of death.In an attempt to overcome this, a recent individual patient meta-analysis showed that the attributable mortality due to VAP was mainly associated with intermediate-severity underlying disease[13]. The relationship of VAT and mortality is even less clear, with some studies showing no deleterious effect on mortality [5, 14] . Nevertheless, a survey of physicians from 288 ICU’s showed that 50% of physicians considered VAT to be associated with increased mortality [4].

There are fewer data concerning the occurrence of VARI in resource-restricted settings but it appears to be at least as common, if not more so, than in resource-rich settings [15–20]. However, estimating its true incidence is difficult and current surveillance definitions have been criticized as particularly inapplicable in resource-restricted settings[21]. VARI occurring in these locations is generally assumed to have similar impact to that occurring in high-income countries but there are limited data to confirm this and some studies have indicated significantly worse outcomes [17, 22]. Differences in underlying diagnoses, age, comorbidity and nutritional status may all influence outcome in addition to variations in treatment facilities and staffing levels [23]. Studies suggest that in low and middle income countries (LMICs), infections are usually caused by multi-drug-resistant Gram-negative organisms [24, 25]. Some studies, although not all, suggest poorer outcome in these patients [17, 26, 27]. This combination of high rates of incidence and antibiotic resistance means VARI may also have an important influence on antibiotic use. A recent study from Viet Nam reported that VARI is the most common reason for antibiotic use in ICU [16].

Understanding the nature and impact of VARI in low-resource settings is especially important as these are places where implementation of relatively simple preventative interventions could have a significant impact [17, 23]. In many ICUs high rates of carbapenem-resistant pathogens necessitate increasing use of empiric regimes including colistin [16]. Identifying factors associated with antimicrobial resistance would therefore enable more effective prevention and treatment measures[17].

In this study we performed a multicenter prospective study following patients receiving mechanical ventilation in four intensive care units in Viet Nam with the aim of providing a comprehensive description of VARI in a resource-restricted setting. We examine the incidence, aetiology and outcome in addition to antibiotic usage and antimicrobial resistance to provide a complete clinical picture of VARI and its impact.

**Methods**

This was a prospective observational study performed in four intensive care units of three tertiary referral hospitals in Viet Nam between November 2013 and November 2015 (total 79 ICU beds). Three sites were ICUs in two specialist infectious disease hospitals: one serving northern Vietnam and the other southern Vietnam (combined capacity 800 beds). The fourth site was a mixed medical and surgical ICU of a 1900-bed teaching hospital. Patients were eligible for study entry if ≥16 years old and expected to be mechanically ventilated for at least 48 hours. Patients intubated for more than 48 hours prior to ICU admission were excluded. An initial sample size of 600 was calculated based on previous data to estimate VAP incidence of 30% with 95% confidence interval of 3.7%[16]. However due to slow recruitment rate, recruitment was stopped after 2 years and enrollment of 450.

Baseline demographic and clinical data were recorded on admission to the study and an endotracheal aspirate was taken as previously described [28]. Patients were followed for 28 days for development of VARI in two sites and until extubation in the remaining sites. Hospital outcome was recorded (death or discharge/ transfer). Clinically suspected VAP was investigated with chest X-ray, and endotracheal aspirate. VAP was diagnosed according to modified US Centres for Disease Control and Prevention (US CDC) criteria [29–31] as follows. Firstly, a deterioration in ventilation following a period of stability defined according to positive end expiratory pressure (PEEP):≥2 days of stable or decreasing daily minimum PEEP followed by a rise in daily minimum PEEP of ≥3 cm H_2_O, sustained ≥2 calendar days; or F_i_O_2_: ≥2 days of stable or decreasing daily minimum F_i_O_2_ followed by a rise in daily minimum F_i_O_2_ ≥0.15 points, sustained ≥2 calendar days. Secondly, systemic signs of fever >38°C or <36°C or white blood cell count >12x10^9/^L or <4x10^9^/L were required. Final criteria were increased/new purulent tracheobronchial secretions or ≥25 neutrophils per low power field (10 objective) on Gram stain of endotracheal aspirate and either new and persistent infiltrates, consolidation, or cavitation as read by two study physicians on chest X-ray, or the decision to commence new antibiotic therapy. VARI was defined as including those patients with VAP (defined as above) and patients meeting the following criteria (modified from Craven et al [8]): clinically increased sputum volume or purulent sputum by microscopic examination (≥25 neutrophils per low power filed on Gram stain of endotracheal aspirate) and either systemic signs of infection (fever or white blood count as above) in addition to the clinician starting antibiotics within 2 days of these features developing. Thus for the purposes of this paper we use the term ‘VAP’ to refer to patients fitting the above definition of VAP; ‘VARI’ to describe all patients with either VAP or VARI as defined above; ‘VARI without VAP’ to refer to patients with VARI as above but not meeting full criteria for VAP. It can be considered that VARI would therefore encompass all patients with VAP and ventilator associated tracheobronchitis (VAT).

The microbiological cause of VARI was determined by isolating at least one pathogenic organism from blood culture or from endotracheal aspirate ≥10^5^ colony forming units/ml or equivalent semi-quantitative culture. Microbiological methods varied by site in line with routine clinical microbiological work. Briefly, in all sites the BACTEC (Becton Dickinson, Sparks, MD, USA) automated system for blood cultures was used with a single aerobic culture bottle inoculated in all suspected cases. Endotracheal aspirate samples were subjected to Ziehl-Neelsen and Gram staining prior to incubation on sheep blood in blood agar base (BioMérieux, Marcy l’Étoile, France), MacConkey and chocolate blood agar. Colonies were identified using routine biochemical testing (involving VITEK2 and/or API test (BioMérieux) or MALDI-TOF MS (Bruker Daltonics, Bremen, Germany)). Antimicrobial susceptibility testing was carried out by the Kirby/Bauer disc diffusion methods, including double disk diffusion for detection of extended spectrum beta-lactamases (ESBL), using cut-offs as per CLSI 2012 guidelines or using VITEK2 depending on site. In addition, CHROMagar (CHROMagar , Paris, France) for detection of methicillin resistant *Staphylococcus aureus* (MRSA), ESBLs, *Amp*C beta-lactamase, and KPC carbapenemase were used in some sites.

Data were recorded onto a case record form and entered into a secure database. Data analysis used Stata Statistical Software, release 8 (Stata Corp LP, College Station, TX USA). Data are given as median (interquartile range (IQR)). Comparison of medians was performed using Mann Whitney U test. Tests of proportion of categorical variables were carried out using chi- squared test. Days at risk of VARI were calculated as days of ventilation and limited to 28 days after enrollment for those not followed for VARI after 28 days. Risk of in-hospital death following VARI was assessed using Cox’s Proportional Hazards Model; those followed for VARI only until 28 days after enrollment were censored at 28 days. Patients with missing hospital discharge data were excluded from this analysis; those with missing APACHE_II data were allocated mean scores depending on admission diagnosis. Logistic regression models were used to evaluate the relationship between admission diagnosis and risk of VARI and relationship with antibiotic treatment. Relationship of ventilation length and admission diagnosis was assessed using Mann Whitney U test.

Antibiotic use was assessed using days of therapy (DOT) defined as the sum of all days on each antibiotic per 100 patient days. For the purpose of this analysis only antibiotics for systemic use were included (i.e. topical preparations were not) and both metronidazole, which is used for treatment of tetanus, and anti-tuberculosis medications were excluded. Antibiotic therapy was defined as “adequate” if the pathogen showed *in vitro* susceptibility, and “inadequate” if it showed intermediate or resistant results. Samples with no significant growth were excluded from these analyses. Cox models assessing effect of antibiotic resistance or adequacy of empiric therapy were constructed using time from VARI as time-to-event variable.

Economic analysis was limited to direct medical costs, derived from hospital bills and based on fees charged to the National Health Insurance and to the patients themselves combined.

**Results**

450 patients were enrolled between November 2013 and November 2015. One patient withdrew from the study and 75 patients were excluded from analyses: 69 were ventilated for less than 48 hours and 6 patients were transferred for extra-corporeal membrane oxygenation (ECMO). Thus, data from 374 patients were available for analysis.

A total of 37/374 (9.9%) patients were diagnosed with VAP with a total incidence density of 8.7/1000 ventilator days. A further 55/374 cases (14.7%), not fulfilling VAP criteria, were diagnosed with VARI, giving a total of 92/374 (24.6%) cases of VARI and an incidence density of 21.7/1000 ventilator days for all VARI. Median time from start of ventilation to developing VAP was 10 days (IQR 5.5-12) and 9 days (IQR 6-15) for cases of VARI without VAP.

*Risk factors for VARI*

Baseline data for patients are given in Tables 1. Baseline data by site are provided in Supplementary Table 1. Patients with both VAP and VARI without VAP experienced longer durations of mechanical ventilation than patients with no VARI. Median duration of ventilation for those with VAP was 22 days (IQR 17-31), 21 days with VARI without VAP (IQR 14-28 days), compared to 10 days (IQR 6-16) with no VARI (p<0.01 for both) (Table 2 and Supplementary Table 2).

To identify baseline predictors of all VARI, admission diagnosis was examined. As this was significantly associated with risk of VARI (OR 1.23 95%CI 1.05-1.45 p=0.01), different admission diagnoses were investigated. Patients with community-acquired pneumonia had the lowest risk of VARI with a risk of 9.6% (95% CI 3-17%). Compared to these, there was a significantly increased risk of VARI in patients with an admission diagnosis of tetanus (36.4%, 95% CI 28.2-45.2%, p<0.01) or ‘other diagnosis’ category (26.7%, 95% CI 14.6-41.9%, p=0.02) (Supplementary table 3). Those with tetanus also had significantly longer duration of mechanical ventilation at a median 18 (IQR 13-24) days and lowest antibiotic use on admission compared to those with community-acquired pneumonia (p<.01 both) (Supplementary table 4).

*Impact of VARI on outcomes*

Patients with VARI experienced longer hospital and ICU stay, increased hospital and ICU cost and increased antibiotic use in the first 28 days of study. This was true for both VAP and VARI without VAP (Table 2) (p<0.01 all).

Compared to patients with no VARI, there were no significant differences in risk of in-hospital death with VARI (HR 0.88, 95% CI 0.44-1.73, p=0.70). When VAP and VARI without VAP subgroups were analyzed, there was a trend towards higher risk of in-hospital death in patients with VAP compared to those with no VARI (HR 1.58, 95% CI 0.75-3.33, p=0.23). However there was a lower risk of in-hospital death in patients with VARI without VAP compared to those with no VARI (HR 0.40, 95% CI 0.14-1.17, p=0.09) which was non-significant. After correction for APACHE II scores, these trends remained non- significant: VAP HR 1.67, 95% CI 0.78—3.55, p=0.18nd VARI without VAP HR 0.46, 95% CI 0.15- 1.39, (p=0.17).

Due to the high proportion of patients with tetanus and its possible confounding, the effect of tetanus on the impact of VARI was specifically examined. Similar to the whole-sample analysis, in both patients with and without tetanus, patients with VARI experienced increased hospital and ICU length of stay, increased duration of ventilation, increased hospital and ICU costs and increased antibiotic use within the 28-day study period (Supplementary Tables 5 & 6.). When tetanus status, Apache II and interaction between tetanus and VARI were included in the cox model, there were no differences in the risk of in-hospital death following either VAP or other VARI (VAP: HR 1.79, 95% CI 0.84-3.82, p=0.13; VARI without VAP (HR 0.43, 95% CI 0.13-1.38, p=0.15).

*Microbiology of VARI*

Of the 92 patients treated for VARI, 77 had endotracheal aspirates taken within 48 hours of VARI diagnosis, 73 of which had positive cultures (Table 3). The predominant bacterium was *Acinetobacter baumannii* (32 patients) followed by *Klebsiella pneumoniae* (26 patients) and *Pseudomonas aeruginosa* (24 patients). 68/73 (92%) patients had specimens containing one or more of these three organisms.

*Antibiotic resistance and impact on outcomes*

New antimicrobial therapy given within 48 hours of VARI diagnosis is given in Supplementary Table 7. The majority of cases (49/92, 54.3%) were treated with a carbapenem; colistin was used in 25/92 (27.2%) cases and combination therapy was used in 36/92 (39.1%) cases. Adequacy of empiric initial antimicrobial therapy of VARI could be assessed in 71 cases and was inadequate in 20/71 (28%) cases. However, inadequate therapy was not associated with increased mortality (HR 0.1.11, 95% CI 0.34-3.66, p=0.86); nor were there differences in length of ICU stay or ICU costs (Table 4). Of note, all of the specimens from patients treated with inadequate antibiotic contained one or more carbapenem-resistant bacteria.

5/10 (50%) *Staphylococcus aureus* isolates were methicillin-resistant. 11/24 (46%) of *P. aeruginosa* isolates and 27/32 (84%) *A. baumannii* isolates were resistant to carbapenems, compared to only 6/26 (23%) of *K. pneumoniae*. In total 40/68 (59%) patients with positive culture for *P. aeruginosa, A. baumannii* or *K. pneumoniae* had carbapenem-resistant isolates. Patients with VARI attributed to these carbapenem-resistant bacteria had significantly greater ICU costs than patients with non-resistant isolates (6053 USD (IQR 3807-7824) vs 3131 USD (IQR 2109-7552), p=0.04). There were no differences between length of hospital or ICU stay but patients with carbapenem-resistant isolates had increased antibiotic use during the study period (37.5 (IQR 29.0-45.5) days of therapy (DOT) compared to 21 (IQR 15.5-33.0) DOT, p<0.01) (Table 5). Additionally, patients with carbapenem-resistant isolates showed a trend towards increased in-hospital death (HR 2.82, 95% CI 0.75-6.75, p=0.15) which remained when adequacy of initial empiric antibiotic therapy and APACHE II scores were taken into account (HR 2.82, 95% CI 0.87-9.19, p=0.09).

In order to assess potential risk factors for development of VARI with carbapenem-resistant bacteria, treatment with carbapenems in ICU before diagnosis of VARI was examined. 22/39 (56%) patients with carbapenem-resistant isolates of *P. aeruginosa, A. baumannii* or *K. pneumoniae* were treated with carbapenems between ICU admission and development of VARI, compared to only 2/26 (7.7%) of those with sensitive isolates (p<0.01).

Discussion

This study has shown that VARI is a common and important problem in resource-restricted ICUs and patients with VARI have an increased length of ICU stay, ICU cost and antibiotic use. Calculating attributable mortality due to VARI is difficult due to difficulty identifying a control group and the numerous possible confounders. By using survival analysis we aimed to account for differences in time at risk. In addition, by including a large subgroup of patients with tetanus we have gained a unique insight into the impact of VARI on patient outcome. Patients with tetanus have little comorbidity or organ dysfunction in addition and they lack features such as increased pulmonary vascular permeability that may confuse the diagnosis of VARI. In these patients we observed increased risk of VARI but very low mortality, and there was no evidence that risk of in-hospital death following VARI differed between patients with or without tetanus.

Although we did not observe any increased risk of in-hospital death in patients with either VAP or VARI without VAP, there was a trend towards improved outcome in patients with VARI without VAP and worse outcome in VAP. Similar findings have previously been reported in different populations. A large study of 2960 patients in Europe and South America reported mortality rates of 40% with VAP, 29% with VAT and 30% with no VARI [5]. Similarly a smaller observational study of patients in a single centre in France reported a mortality rate of 29% in VAT patients compared to 36% of controls without VARI [14]. VARI may be viewed as a continuum between VAT and VAP both in terms of severity and chronology: i.e. there is a progression from asymptomatic bacterial colonization of the respiratory tract through tracheobronchitis and eventually to pneumonia when chest X-ray changes become apparent [8, 32]. It is possible that patients meeting the full criteria for VAP have more severe disease, are diagnosed later and therefore have worse outcome. This is supported by recent evidence that patients with VAP treated early with antibiotics showed higher response rates [33].

In 2013, as this study was being conceived, the CDC introduced a system of ventilator associated event definitions, which encompassed criteria for ventilator associated pneumonia. Our choice of definitions for use in this study was influenced by a desire to balance relevant, objective definitions with these new definitions. These definitions have now been widely criticized and it has been recognized that they may need to be adjusted for specific patient groups [34]. The CDC explicitly states that some elements of the new framework (probable VAP) are not suitable for public reporting or bench-marking [35]. For this reason we chose to use the definitions we felt best reflected clinical practice and were relevant to the main issues of this study, namely the high use of broad-spectrum antibiotics in resource-restricted ICUs and outcome of patients treated for VARI. By using clear and consistent definitions throughout we aimed to minimize bias when comparing these outcomes. As our main aim was to describe actual practice in resource-limited ICUs, and 3 out of 4 participating ICUs in our study were specialist infectious disease units we deliberately chose to include patients with infections, including an admission diagnosis of pneumonia. An earlier pilot study had shown that not including patients with a possible pneumonia on admission led to patients with other primary diagnose such as sepsis or meningitis being excluded from the study due to abnormal baseline chest x-rays. In view of this we felt excluding these patients would introduce significant bias and reduce the relevance of this study for future practice.

Overall, compared to other studies in resource-restricted settings, we recorded a low incidence of VAP, but comparable levels of total VARI [16, 26, 36]. The low rate of VAP may be due to the insensitivity of VAP criteria used in our setting resulting in possible misclassification of cases. In our ICUs, low staffing levels, lack of ICU-specific training and less frequent monitoring mean that higher ventilator settings are often used and ventilator settings are infrequently changed to reduce the risk of hypoxia. Thus the demonstrable deterioration in ventilator settings (ie PEEP or F_i_O_2_) to diagnose VAP may be less likely to occur resulting in possible VAP cases classified as VARI without VAP. However, a strength of our study is that we have included all patients with VARI, thus patients not meeting VAP criteria, but treated for ventilator-related respiratory infection have still been included in the analyses. The differences in VAP rates between ICUs included in our study may reflect differences in practices between ICUs as the overall VARI rates are similar.

Unlike VARI described in high-income settings, isolates associated with VARI were dominated by Gram-negative bacteria [37]. Of note, 92% of our patients had VARI attributable to one or more of the World Health Organization’s three ‘critical’ priority organisms: *K. pneumoniae*, *P. aeruginosa* and *A. baumannii* [38]. We report high levels of antimicrobial resistance among these and also note that resistance to carbapenems was associated with a worse outcome and that prior use of carbapenems during hospital admission was a risk factor for isolation of carbapenem resistant bacteria, suggesting a role for carbapenem-sparing agents in the management of severe infection. Furthermore, carbapenem resistance had a significant and substantial impact on costs and antibiotic use.

The lack of correlation between inadequate empiric therapy and mortality is in keeping with some of the literature from high income countries, much of which comes from studies in sepsis and septic shock, yet is out of step with other studies [38–40]. In the context of this study it most likely also relates to the behaviour of clinicians faced with a single remaining option of colistin for treatment of increasingly resistant Gram-negative infections; reserving empiric treatment more likely to be adequate (i.e. colistin) for more severe cases. We have only analysed the effects of adequate and inadequate therapy with respect to initial empiric therapy and it is also possible that timely reporting of microbiological culture results enabled rapid rectification of inadequate regimes hence limited impact on outcome measures.

Patient hospitalization costs were significantly and substantially higher in patients with VARI and approximately double for those with carbapenem-resistant organisms. In our setting, antibiotics such as carbapenems or colistin form a higher proportion of ICU cost than in resource-rich settings and treatment regimens for multidrug resistant organisms include treatment with more expensive antibiotics for longer periods. A limitation of our study is that we have not been able to divide healthcare costs before and after VARI and as such may be subject to immortal time bias. Furthermore, we have only analyzed direct patient treatment costs and have not taken account of additional healthcare costs of staffing and equipment or indirect costs such as lost earnings, travel and subsistence costs of patients and family members providing care. In a resource-restricted setting such as ours, where much of hospital care is provided by family members, these costs are likely to be substantial and an important additional burden.

Despite these limitations, we believe VARI remains an important problem in resource-restricted ICUs and clinicians working in these settings need locally-relevant evidence demonstrating the efficacy and safety of interventions to reduce its incidence. Previous work aiming to prevent infection through patient positioning and hand-hygiene have not shown benefit in our setting [41, 24], emphasizing the need for locally derived data. To this end we are engaged in a randomized controlled trial of continuous cuff pressure to prevent ventilator associated respiratory infections (clinicaltrials.gov idn NCT02966392).

Conclusions

VARI is a significant problem in a resource-restricted ICU setting. Despite no observed impact on mortality, there are significant and substantial cost implications related to occurrence of VARI, particularly that associated with carbapenem-resistant bacteria. Dealing with these high rates of carbapenem resistance needs to involve both antibiotic stewardship and infection prevention strategies.

**List of abbreviations**

CI Confidence interval

DOT Days of therapy

ECMO extra-corporeal membrane oxygenation

ESBL Extended spectrum beta lactam

FiO2 Fraction of inspired oxygen

HR Hazard ratio

ICU Intensive care unit

IQR Interquartile range

LMIC low and middle income country

PEEP Positive end expiratory pressure

USD United States dollar

VAP Ventilator associated pneumonia

VARI Ventilator associated respiratory infection

VAT Ventilator associated tracheobronchitis

**Declarations**

Ethics approval and consent to participate

The study was approved by the Oxford Tropical Research Ethics Committee and local Ethics Committees of participating hospitals (Scientific and Ethics Committees of Bach Mai Hospital Hanoi, National Hospital for Tropical Diseases Hanoi and Hospital for Tropical Diseases, Ho Chi Minh City). The study was carried out according to the principals of the Declaration of Helsinki and all participants gave written informed consent to take part in the study.

Consent for publication

All authors have seen and approved the final manuscript for publication.

Availability of data and material

OUCRU pursues a Data Sharing policy in collaboration with the Hospital for Tropical Diseases Ho Chi Minh City and the National Hospital for Tropical Diseases Hanoi. Data from the study are available following application to the corresponding author.

In addition data are available from the Oxford Research Archive 9http://researchdata.ox.ac.uk/

Competing interests

None

Funding

This study was funded by the Wellcome Trust UK, Swedish International Development Cooperation Agency (SIDA) and the Li Ka Shing Foundation

Authors' contributions

VDP was involved in conception/design, data acquisition, analysis/interpretation, drafting/revising the manuscript. BN was involved in conception/design, data acquisition, analysis/interpretation and drafting/revising the manuscript. DZC was involved in conception/design and data acquisition. NHAD was involved in conception/design, data acquisition and analysis/interpretation. NTHM was involved in conception/design, data acquisition and analysis/interpretation. NTT was involved in data acquisition. JC was involved in conception/ design and data acquisition. DPK was involved in data acquisition. TNQ was involved in data acquisition. HTL was involved in conception/design and data acquisition. DSB was involved in in conception/design and data acquisition. QDD was involved in conception/ design and drafting/revising the manuscript. DBT was involved in data acquisition and revising the manuscript. HL was involved in data acquisition. NHH was involved in conception/ design. AB was involved in data acquisition and revising the manuscript. ML was involved in conception/ design and drafting/ revising the manuscript. MHM was involved in conception/ desin and data acquisition. GQT was involved in data acquisition. LTDT was involved in data acquisition. GTA was involved in data acquisition. HH was involved in conception/ design. HNMV was involved in data acquisition. RBG was involved in data analysis/ interpretation and drafting/ revising the manuscript. LMY was involved in conception/ design and data acquisition. NVH was involved in conception/ design and data acquisition. NGB was involved in conception/ design and data acquisition. NVVC was involved inconception/ design. NVK was involved in conception/ design. GET was involved in conception/ design and drafting/ revising the manuscript. HRW was involved in conception/ design/ data acquisition, analysis and drafting/ revising the manuscript. HRVD was involved in conception/ design/ data acquisition, analysis and drafting/ revising the manuscript. CLT was involved in conception/ design/ data acquisition, analysis and drafting/ revising the manuscript.

Acknowledgements

We would like to thank staff in the Biostatistics and Oxford University Clinical Research Unit, Centre for Tropical Medicine and Global Health, University of Oxford for help and statistical advice.

We also thank all staff in ICUs at the National Hospital for Tropical Diseases, Hospital for Tropical Diseases and Bach Mai Hospitals for their help with this study as well as the Clinical Trials Unit and Data Management Group at Oxford University Clinical Research Unit Ha Noi and Ho Chi Minh City.

References

1. Vincent J-L, Sakr Y, Sprung CL, et al. Sepsis in European intensive care units: results of the SOAP study. Crit Care Med 2006; 34:344–353.

2. Zarb P. The European Centre for Disease Prevention and Control ( ECDC ) pilot point prevalence survey of healthcare-associated infections and antimicrobial use The European Centre for Disease Prevention and Control ( ECDC ) pilot point prevalence survey of health. Euro surveil 2012; 17:pii: 20316.

3. Dudeck MA, Weiner LM, Allen-Bridson K, et al. National Healthcare Safety Network (NHSN) report, data summary for 2012, Device-associated module. Am J Infect Control 2013; 41:1148–1166.

4. Rodríguez A, Póvoa P, Nseir S, et al. Incidence and diagnosis of ventilator-associated tracheobronchitis (VAT) in the intensive care unit: an international online survey. Crit Care 2014; 18:R32.

5. Martin-Loeches I, Povoa P, Rodríguez A, et al. Incidence and prognosis of ventilator-associated tracheobronchitis (TAVeM): a multicentre, prospective, observational study. Lancet Resp Med 2015; 3:859–68.

6. European Centre for Disease Prevention and Control. Surveillance of healthcare-associated infections in Europe 2007. Stock ECDC 2012. doi: 10.2900/18553

7. Waltrick R, Possamai D, Perito de Aguair F, et al. Comparison between a clinical diagnosis method and the surveillance technique of the Center for Disease Control and Prevention for identification of mechanical ventilator-associated pneumonia. Rev Braz Ter Intensiva 2015; 27:260–265.

8. Craven DE, Lei Y, Ruthazer R, et al. Incidence and outcomes of ventilator-associated tracheobronchitis and pneumonia. Am J Med 2013; 126:542–9.

9. Murray CJL, Vos T, Lozano R, et al. Disability-adjusted life years (DALYs) for 291 diseases and injuries in 21 regions, 1990-2010: a systematic analysis for the Global Burden of Disease Study 2010. Lancet 2012; 380:2197–223.

10. Klompas M. Is a ventilator-associated pneumonia rate of zero really possible? Curr Opin Infect Dis 2012; 25:176–82.

11. Restrepo MI, Anzueto A, Arroliga AC, et al. Economic Burden of Ventilator-Associated Pneumonia Based on Total Resource Utilization. Infect Control Hosp Epidemiol 2010; 31:509–15.

12. Shahin J, Bielinski M, Guichon C, et al. Suspected ventilator-associated respiratory infection in severely ill patients: a prospective observational study. Crit Care 2013; 17:R251.

13. Melsen, Wilhelmina G Rovers MM, Groenwold RHH, Bergmans, Dennis C J J Camus C, et al. Attributable mortality of ventilator-associated pneumonia : a meta-analysis of individual patient data from randomised prevention studies. Lancet 2013; 13:665–671.

14. Nseir S, Di Pompeo C, Soubrier S, et al. Effect of ventilator-associated tracheobronchitis on outcome in patients without chronic respiratory failure: a case-control study. Crit Care 2005; 9:R238-45.

15. World Health Organization. The Burden of Health Care-Associated Infection Worldwide A Summary 2004. http://www.who.int/gpsc/country_work/summary_20100430_en.pdf

16. Phu VD, Wertheim HFL, Larsson M, et al. Burden of hospital acquired infections and antimicrobial use in Vietnamese adult intensive care units. PLoS One 2016 11:1–15. doi: 10.1371/journal.pone.0147544

17. Arabi Y, Al-shirawi N, Memish Z, Anzueto A. Ventilator-associated pneumonia in adults in developing countries : a systematic review. Int J Infec Dis 2008:505–12.

18. Bammigatti C, Doradla S, Narashimha B, et al. Healthcare Associated Infections in a Resource Limited Setting. J Clin Diagn Res 2017; 11:5–8.

19. Salgado Yepez E, Bovera MM, Rosenthal VD, et al. Device-associated infection rates, mortality, length of stay and bacterial resistance in intensive care units in Ecuador: International Nosocomial Infection Control Consortium’s findings. World J Biol Chem 2017; 8:95.

20. Ray U, Ramasubban S, Chakravarty C, et al. A prospective study of ventilator-associated tracheobronchitis:Incidence and etiology in intensive care unit of a tertiary care hospital. Lung india 2017; 34:236–40.

21. Rrt HC, Chen C, Rrt SK, et al. American Journal of Infection Control Differences between novel and conventional surveillance paradigms of ventilator-associated pneumonia. Am J Infect Control 2015; 43:133–136.

22. Rosenthal VD, Maki DG, Salomao R, et al. Device-associated nosocomial infections in 55 intensive care units of 8 developing countries. Ann Intern Med 2006; 145:582–591.

23. Schultz MJ, Dunser MW, Dondorp AM, et al. Current challenges in the management of sepsis in ICUs in resource-poor settings and suggestions for the future. Intensive Care Med 2017. doi: 10.1007/s00134-017-4750-z

24. Schultsz C, Bootsma MCJ, Loan HT, et al. Effects of infection control measures on acquisition of five antimicrobial drug-resistant microorganisms in a tetanus intensive care unit in Vietnam. Intensive Care Med 2013; 39:661–71

25. Inchai J, Pothirat C, Liwsrisakun C, et al. Ventilator-associated pneumonia: epidemiology and prognostic indicators of 30-day mortality. Jpn J Infect Dis 2015; 68:181–6.

26. Mathai AS, Phillips A, Isaac R. Ventilator ‑ associated pneumonia : A persistent healthcare problem in Indian Intensive Care Units ! Lung India 2016; 33:512–516.

27. Blot S, Koulenti D, Dimopoulos G, et al. Prevalence, Risk Factors, and Mortality for Ventilator-Associated Pneumonia in Middle-Aged, Old, and Very Old Critically Ill Patients*. Crit Care Med 2014; 42:601–609.

28. Loan HT, Parry J, Nga NTN, et al. Semi-recumbent body position fails to prevent healthcare-associated pneumonia in Vietnamese patients with severe tetanus. Trans R Soc Trop Med Hyg 2012; 106:90–7.

29. Centers for Disease Control and Prevention (CDC) Pneumonia (Ventilator-associated [VAP] and non-ventilator-associated Pneumonia [PNEU]) Event. http://www.cdc.gov/nhsn/pdfs/pscmanual/6pscvapcurrent.pdf

30. Klompas M, Kleinman K, Khan Y, et al. Rapid and reproducible surveillance for ventilator-associated pneumonia. Clin Infect Dis 2012; 54:370–7.

31. Johanson W, Pierce A, Sanford J, Thomas G. Nosocomial respiratory infections with gram-negative bacilli. The significance of colonization of the respiratory tract. Ann Int Med 1971; 77:701–6.

32. Craven DE, Hudcova J, Lei Y, et al. Pre-emptive antibiotic therapy to reduce ventilator-associated pneumonia : “ thinking outside the box .” Crit Care 2016; 20:300–12.

33. Ramirez P, Lopez-ferraz C, Gordon M, et al. From starting mechanical ventilation to ventilator-associated pneumonia , choosing the right moment to start antibiotic treatment. Crit Care 2017; 1–7. doi: 10.1186/s13054-016-1342-1

34. Spalding MC, Minshall CT. Ventilator-Associated Pneumonia: New Definitions. Crit Care Clin 2017; 33:2:277-292.

35. Klompas M, Berra L. Should Ventilator-Associated Events become a Quality Indicator for ICUs? Respir Care 2016; 61:723–736.

36. Allegranzi B, Nejad SB, Combescure C, et al. Burden of endemic health-care-associated infection in developing countries : systematic review and meta-analysis. Lancet 2011; 377:228–241.

37. Chastre J, Fagon J-Y. Ventilator-associated Pneumonia. Am J Respir Crit Care Med 2002; 165:867–903.

38. WHO (2017) Global Priority List of Antibiotic-Resistant Bacteria To Guide Research, Discovery, and Development of New Antibiotics. http://www.who.int/medicines/publications/WHO-PPL-Short_Summary_25Feb-ET_NM_WHO.pdf

39. Swanson J, Wells D. Empirical Antibiotic Therapy for Ventilator-Associated Pneumonia. Antibiotics 2013; 2:339–351.

40. Thwaites CL, Lundeg G, Dondorp AM. Recommendations for infection management in patients with sepsis and septic shock in resource-limited settings. Intensive Care Med 2016. doi: 10.1007/s00134-016-4415-3

41. Thwaites CL, Yen LM, Nga NTN, et al. Impact of improved vaccination programme and intensive care facilities on incidence and outcome of tetanus in southern Vietnam, 1993-2002. Trans R Soc Trop Med Hyg2004; 98:671–677.

Table 1. Baseline characteristics of patients with and without VARI

|  | All VARI (n=92) | VAP (n=37) | VARI without VAP (n=55) | No VARI (n=286) |
| --- | --- | --- | --- | --- |
| Age | 48 (36-61.5) | 46 (37-62) | 50 (35-62) | 53 (40-65) |
| M:F | 70:22 | 25:12 | 45:10 | 197:85 |
| SOFA | 3 (2-6) | 5 (2-8) | 3 (1-3) | 4 (3-7) |
| APACHE | 8.5 (5-14) | 11 (5-15) | 7 (5-14) | 11 (7-15) |
| BMI | 21.97 (19.72-24.56) | 22.12 (21.12-24.61) | 21.97 (19.03-23.53) | 20.56 (18.69-23.48) |
| Comorbidity | 26 (28.3%) | 14 (37.9%) | 12 (21.8%) | 118 (42.1%) |
| Hospital previous 90 days† | 7 (7.6%) | 3 (8.1%) | 4 (7.3%) | 34 (12.1%) |
| Transferred for this illness | 69 (75%) | 27 (73.0%) | 42 (76.4%) | 212 (75.18) |
| Antibiotics in last 90 days | 4 (4.4%) | 3 (8.1%) | 1 (1.8%) | 21 (7.5%) |
| CNS infection | 11 (12.0%) | 1 (2.7%) | 10 (18.2%) | 49 (17.4%) |
| Dengue | 3 (3.3%) | 2 (5.4%) | 1 (1.8%) | 6 (2.1%) |
| Sepsis/septic shock | 11 (12.0%) | 6 (16.2%) | 5 (9.1%) | 44 (15.6%) |
| Tetanus | 48 (52.2%) | 14 (37.8%) | 34 (61.8%) | 84 (29.8%) |
| Pneumonia | 7 (7.6%) | 7 (18.9%) | 0 (0%) | 66 (23.4%) |

†n=249

Values given are median (IQR) or count (percent)

Table 2: Outcome of patients with VARI,VAP, Other VARI and no VARI

|  | All VARI (n=92) | VAP (n=37) | VARI without VAP (n=55) | No VARI (n=286) |
| --- | --- | --- | --- | --- |
| ICU Length of stay (days) | 27 (21-37) | 25 (19-37) | 27 (25-38) | 16 (10-28) |
| Days Ventilated (days) | 22 (15.5-29.5) | 21 (14-28) | 22 (17-31) | 10 (6-16) |
| ICU cost (USD) * | 4723 (2778-7783) | 7213 (3011-8329) | 4196 (2726-7193) | 2534 (1493-4407) |
| Hospital Length of stay (days) | 38 (26-49) | 31 (22-46) | 39.5 (29.5-49) | 25 (14-39) |
| Hospital cost (USD)** | 4434 (2656-7822) | 4522 (2716-7990) | 4167 (2658-6985) | 2639 (1555-4576) |
| Antibiotics use (DOT) | 31 (18-42.5) | 35 (21-45) | 30 (17-38) | 18 (10-28) |
| Antibiotics in hospital before VARI | 73/92 (79.4%) | 28/38 (75.6%) | 45/55 (87.7%) | 255/282 (90.43%) |
| In-hospital mortality | 17/91 (18.7%) | 13/37 (35.1%) | 4/54 (7.4%) | 54/277 (19.5%) |

* n= 372; ** n=313

Values given are median (IQR) or count (percent)

Table 3 Organisms isolated from initial endotracheal aspirates within 48 hours of VARI diagnosis

|  | N (% isolates) | Carbapenem resistance N (%) |
| --- | --- | --- |
| *Acinetobacter baumannii* | 32 (29.7%) | 27/32 (84.4%) |
| *Klebsiella pneumoniae* | 26 (24.1%) | 6/26 (23.1%) |
| *Pseudomonas aeruginosa* | 24 (22.2%) | 11/24 (45.8%) |
| *Staphylococcus aureus* | 10 (9.3%) | 5* (50%) |
| *Haemophilus influenza* | 5 (4.6%) | 0 (0%) |
| *Elizabethkingia meningoseptica* | 3 (2.8%) | 1 (33.3%) |
| *Stenotrophomonas maltophilia* | 3 (2.8%) | 3 (100%) |
| *Streptococcus pneumoniae* | 3 (2.8%) | 0* (0%) |
| *Escherichia coli* | 1 (0.9%) | 1 (100%) |
| *Proteus mirabilis* | 1 (0.9%) | 0 (0%) |

*Carbapenem susceptibility not formally assessed but carbapenem susceptibility assumed from penicillin or methicillin sensitivity.

Table 4 Adequacy of Initial Empiric Antibiotic Therapy for VARI

|  | Adequate | Inadequate | P |
| --- | --- | --- | --- |
| In-hospital mortality | 9/51 | 4/20 | 1.00 |
| ICU length of stay (days) | 27 (22-37) | 27.5 (25-37) | 0.74 |
| Hospital length of stay (days) | 38 (26-49) | 39 (28.5-44) | 0.89 |
| ICU cost (USD) | 4797 (2726-7806) | 5485 (3541-7259) | 0.88 |
| Hospital cost (USD) | 4461 (2666-7883) | 5549 (3843-7543) | 0.54 |

Values given are median (IQR) or count (percent)

Table 5 Impact of Carbapenem-resistant Bacteria causing VARI

|  | Carbapenem resistant Enterobacteriaceae/*A. baumannii* or *P. aeruginosa* | Carbapenem sensitive Enterobacteriaceae/*A. baumannii* or *P. aeruginosa* | p |
| --- | --- | --- | --- |
| ICU length of stay (days) | 26 (20.5-33.5) | 29.5 (23.5-39.5) | 0.23 |
| ICU cost (USD) | 6053 (3806-7824) | 3131 (2108-7551) | 0.04 |
| In-hospital mortality | 10/40 (25%) | 3/28 (10.7%) | 0.14 |
| Antibiotic use (DOT) | 37.5 (29-45.5) | 21 (15.5-33) | <0.01 |

Values given are median (IQR) or count (percent)

Supplementary Table 1: Baseline characteristics by site

|  | AICU HTD (n=127) | VA ICU HTD (n=25) | NHTD (n=161) | BM (n=61) |
| --- | --- | --- | --- | --- |
| Age | 49 (34-67) | 51 (27-62) | 52 (42-62) | 56 (42-67) |
| M:F | 93:34 | 16:9 | 116:45 | 42:19 |
| BMI | 20.81 (19.42-23.43) | 20 (19.01-23.85) | 19.14 (17.78-21.37) | 22.41 (19.53-24.91) |
| SOFA | 2 (1-4) | 6 (4-7) | 5 (3-9) | 5.5 (3-7) |
| APACHE II | 7.5 (5-11) | 16 (12-19) | 11 (7-15) | 13 (9.5-17) |
| Transferred this illness | 95 (74.8%) | 22 (88%) | 143 (88.82) | 21 (34.43) |
| Hospital 90 days preceding | 6 (4.72) | 1(4) | 24 (14.91) | 10 (16.39) |
| comorbidity | 18 (14.29) | 7 (28) | 74 (46.25) | 45 (73.77) |
| Tetanus | 96 (75.59) | 0 | 36 (22.36) | 0 |
| Dengue | 9 (7.09) | 0 | 0 | 0 |
| CNS infections | 1 (0.79) | 24 (96) | 34 (21.12) | 1 (1.64) |
| Sepsis/septic Shock | 11 (8.66) | 0 | 38 (23.60) | 6 (9.84) |
| Pneumonia | 7 (5.51) | 0 | 48 (29.81) | 18 (29.51) |
| Other diagnosis | 2 (2.36) | 1 (4) | 5 (3.11) | 36 (59.02)) |

Values given are median (IQR) or count (percent)

Supplementary Table 2: VARI and outcome by site

|  | AICU HTD (n=127) | VA ICU HTD (n=25) | NHTD (n=161) | BM (n=61) |
| --- | --- | --- | --- | --- |
| VAP | 16 (12.6%) | 1 (4%) | 12 (8.16%) | 8 (13.11%) |
| VARI | 36 (28.35%) | 4 (16%) | 39 (24.22%) | 13 (21.31%) |
| ICU Length of stay | 22 (15-29) | 36 (22-51) | 20 (12-34) | 12 (9-18) |
| Days ventilated | 14 (7-21) | 16 (7-33) | 14 (8-25) | 7 (6-12) |
| In-hospital mortality | 17 /127(13.39) | 10/25 (40) | 34/161 (21.12) | 10/55 (18.18) |

Values given are median (IQR) or count (percent)

Supplementary Table 3: Risk of VARI according to admission diagnosis

|  | Cases VARI (%) | Odds Ratio (95% CI) | P |
| --- | --- | --- | --- |
| Pneumonia | 7/73 (9.59) | reference |  |
| Dengue | 3/9 (33.3) | 4.71 (0.96-23.1) | 0.06 |
| CNS infection | 11/60 (18.3) | 2.11 (0.77-5.99) | 0.15 |
| Tetanus | 48/132 (36.4) | 5.39 (2.29-12.68) | <0.01 |
| Sepsis/ septic shock | 11/55 (20) | 2.35 (0.85-6.55) | 0.10 |
| Other diagnosis | 12/45 (26.7) | 3.431.23-9.52 | 0.02 |

Supplementary Table 4: Duration of ventilation and antibiotic use in patients according to admission diagnosis

| Admission Diagnosis | Duration ventilation (days) median (IQR) | p | Antibiotic treatment on admission to ICU | p |
| --- | --- | --- | --- | --- |
| Pneumonia | 9 (6-16) | Reference | 63/73 (86.3%) | Reference |
| Dengue | 5 (4-8) | 0.03 | 7/9 (77.8%) | 0.50 |
| CNS Infection | 16 (7.5-33) | <0.01 | 46/60 (76.7%) | 0.16 |
| Tetanus | 18 (13-24) | <0.01 | 53/132 (40.1%) | <0.01 |
| Sepsis/Septic shock | 8 (6-13) | 0.39 | 52/55 (94%) | 0.14 |
| Other diagnosis | 7 (5-12) | 0.03 | 27/45 (60%) | <0.01 |

Values given are median (IQR) or count (percent)

Supplementary Table 5 Impact of VARI in patients with and without tetanus

|  | VARI | No VARI | P |
| --- | --- | --- | --- |
| In-hospital mortality in patients without tetanus | 13/43 (30.23%) | 50/193 (25.914%) | 0.56 |
| In-hospital mortality in patients with tetanus | 4/48 (8.33%) | 4/84 (4.76%) | 0.41 |
| ICU Length of stay in patients without tetanus (days) | 24.5 (15.5-32.5) | 13.5 (8-23) | <0.01 |
| ICU Length of stay in patients with tetanus (days) | 29.5 (25-37.5) | 23 (17-30) | <0.01 |
| Hospital Length of stay in patients without tetanus (days) | 26 (19-46) | 18 (11-30) | <0.01 |
| Hospital Length of stay in patients with tetanus (days) | 41 (31.5-49) | 35 (28.5-41) | <0.01 |
| Days ventilated in patients without tetanus (days) | 19 (12-31.5) | 8 (5-13) | <0.01 |
| Days ventilated in patients with tetanus (days) | 23 (18.5-29) | 15 (10-20.5) | <0.01 |
| ICU cost in patients without tetanus (USD)* | 7083 (3451-9146) | 3018 (1747-5494) | <0.01 |
| ICU cost in patients with tetanus (USD) | 3870 (2266-6420) | 1615 (1221-2741) | <0.01 |
| Hospital cost in patients without tetanus (USD)** | 5792 (3053-9429) | 3347 (1883-5596) | <0.01 |
| Hospital cost in patients with tetanus (USD) | 4083 (2281-6523) | 1801 (1338-3030) | <0.01 |
| Antibiotic use in patients without tetanus (DOT) | 34.5 (20.5-53.5) | 22 (12-30) | <0.01 |
| Antibiotic use in patients with tetanus (DOT) | 29.5 (17-37.5) | 12.5 (9-20) | <0.01 |

* n= 242 **n=181

Values given are median (IQR) or count (percent)

Supplementary Table 6. Impact of VARI and other VARI in patients with and without tetanus

|  | VAP | VARI without VAP | No VARI | P^1^ | P^2^ |
| --- | --- | --- | --- | --- | --- |
| In-hospital mortality in patients without tetanus | 11/23 (47.85%) | 2/20 (10.0%) | 50/193 (25.914%) | 0.03 | 0.15 |
| In-hospital mortality in patients with tetanus | 2/14 (14.29%) | 2/34 (5.88%) | 4/84 (4.76%) | 0.17 | 0.80 |
| ICU Length of stay in patients without tetanus (days) | 19 (14-28) | 26 (21-39) | 13.5 (8-23) | <0.01 | <0.01 |
| ICU Length of stay in patients with tetanus (days) | 29 (25-38) | 30 (25-37) | 23 (17-30) | <0.01 | <0.01 |
| Hospital Length of stay in patients without tetanus (days) | 24 (17-36) | 32 (25-49) | 18 (11-30) | 0.02 | <0.01 |
| Hospital Length of stay in patients with tetanus (days) | 41.5 (38-58) | 40.5 (31-45) | 35 (28.5-41) | <0.01 | 0.03 |
| Days ventilated in patients without tetanus (days) | 17 (12-26) | 20 (15-33) | 8 (5-13) | 0.02 | <0.01 |
| Days ventilated in patients with tetanus (days) | 25 (19-31) | 22.5 (18-29) | 15 (10-20.5) | 0.02 | <0.01 |
| ICU cost in patients without tetanus (USD)* | 7760 (3433-11973) | 5981 (3470-7810) | 3018 (1747-5494) | <0.01 | <0.01 |
| ICU cost in patients with tetanus (USD) | 3951 (2645-7730) | 3852 (2124-6400) | 1615 (1221-2741) | <0.01 | <0.01 |
| Hospital cost in patients without tetanus (USD)** | 5118 (2633-10990) | 5939 (3770-8976) | 3347 (1883-5596) | 0.07 | <0.01 |
| Hospital cost in patients with tetanus (USD) | 4340 (2716-7883) | 3933 (2133-6398) | 1801 (1338-3030) | <0.01 | <0.01 |
| Antibiotic use in patients without tetanus (DOT) | 41 (24-54) | 29 (19-37) | 22 (12-30) | <0.01 | 0.02 |
| Antibiotic use in patients with tetanus (DOT) | 27.5 (21-31) | 30.5 (16-38) | 12.5 (9-20) | <0.01 | <0.01 |

1 Comparison of VAP and no VARI

2 Comparison of other (non-VAP) VARI and no VARI

* n= 242 **n=181

Supplementary Table 7 Empiric antibiotic therapy for VARI

| Class of antibiotic | N | % VARI cases |
| --- | --- | --- |
| Carbapenems | 49 | 54.3 |
| Polymixins (colistin) | 25 | 27.2 |
| Fluoroquinolones | 16 | 17 |
| Combinations of penicillins included beta-lactamase inhibitors | 9 | 9.8 |
| 3^rd^ Generation cephalosporins | 7 | 7.6 |
| Other aminoglycosides | 7 | 7.6 |
| Glycopeptides | 6 | 6.5 |
| Other antibacterials | 2 | 2.2 |
| Macrolide antibiotics | 1 | 1.1 |
